# Development of *Mycobacterium tuberculosis *attenuated strains as live vaccine candidates for tuberculosis

**DOI:** 10.1186/1753-6561-8-S4-O5

**Published:** 2014-10-01

**Authors:** Anne Villela, Valnês Rodrigues-Junior, Luiz Augusto Basso, Diógenes Santos

**Affiliations:** 1 Centro de Pesquisas em Biologia Molecular e Funcional (CPBMF), Instituto Nacional de Ciência e Tecnologia em Tuberculose (INCT-TB), PUCRS. Av. Ipiranga, 6681 - TECNOPUC - Prédio 92A Partenon - Porto Alegre, CEP 90619-900, Brazil

## 

Tuberculosis (TB) is an infectious disease caused mainly by the bacillus *Mycobacterium tuberculosis*. The World Health Organization estimates that 8.6 million new TB cases occurred in 2012, resulting in 1.3 million deaths [1]. TB is the second leading cause of death caused by an infectious disease worldwide after immunodeficiency virus (HIV). Despite the availability of an effective chemotherapy and a moderately protective Bacille-Calmette-Guérin (BCG) vaccine, TB remains a major global health problem [1]. BCG provides efficient protection against TB in newborns, but its efficacy against the establishment of latent or pulmonary TB in adults is highly variable. The variability of BCG protection in adults might be related to the absence of more than a hundred genes when compared with the *Mycobacterium bovis *pathogenic strain. Among the missing genes in BCG is the RD1 region which encodes potent antigens and virulence factors [2]. Thus, there is an urgent need for the emergence of new prophylactic strategies to decrease TB incidence worldwide. The development of genetic tools to manipulate mycobacteria and the completion of *M. tuberculosis *genome sequencing have been contributing to a better understanding of genes involved in TB virulence and pathogenesis, and consequently to the emergence of novel vaccine candidates.

Two major strategies have been used to develop new vaccine candidates against TB: (i) substitution of BCG in which an improved version of BCG or a new attenuated live *M. tuberculosis *vaccine would have a higher efficiency than BCG and replace it as a prime vaccine; and (ii) a prime-boost strategy in which BCG continues to be given to neonates, and a new vaccine is given as a booster dose to extend the protection and efficacy [1]. Following these approaches, numerous vaccine candidates against TB are currently in preclinical and clinical trials, including recombinant BCGs, attenuated *M. tuberculosis *strains, recombinant viral-vectored platforms, protein/adjuvants combinations and mycobacterial extracts [1]. The strategy to develop novel vaccines based on the construction of rationally attenuated *M. tuberculosis *strains holds the advantage of potentially eliciting a more sustained protective immune response than viral vectored and recombinant protein candidates.

The successful isolation of allelic exchange mutants of *M. tuberculosis *is dependent on the ability of the genetic tools to enable the efficient detection and selection of mutants among the total population of transformants [3]. Suicide and conditionally-replicating delivery plasmids that combines both selectable and counter-selectable markers, reporter gene, and a mycobacterial thermosensitive origin of replication have been widely used to demonstrate attenuation of *M. tuberculosis *mutant strains [3].

The first step towards the development of a new live vaccine candidate against TB is the evaluation of mutant strain attenuation followed by the investigation of its ability to generate an immune response. Live vaccine candidates have to mimic natural infection as closely as possible without causing disease, in which, a right balance between attenuation and immunogenicity has to be reached, since over attenuated bacteria may not produce *in vivo *some key antigens for the induction of a protective immunity [4].

An efficient strategy to rationally attenuate *M. tuberculosis *consists on the construction of double-deletion mutant of *M. tuberculosis *in the *panC *and *panD *genes, both involved in the *de novo *biosynthesis of pantothenate. Pantothenic acid (vitamin B5) is an essential molecule required for the synthesis of coenzyme A and acyl carrier protein (ACP), two important molecules in fatty acid metabolism and other metabolic reactions [5]. The double-deletion mutant resulted in an auxotrophic and attenuated strain which conferred protection in mice challenged with virulent *M. tuberculosis *[5]. In attempts to further enhance the safety of this live attenuated *M. tuberculosis *vaccine candidate, two other mutant strains were constructed combining *panCD *deletion with either *lysA *or *leuD *deletions, involved in lysine and leucine biosynthesis, respectively. The mc^2^6020 strain, constructed by the inactivation of the *panCD *and *lysA *genes, is strictly auxotrophic for pantothenate and lysine, severely attenuated and capable of inducing protective responses against an aerosolized *M. tuberculosis *challenge in both immunocompetent and immunocompromised mice [6]. The strain constructed by inactivation of the *panCD *and *leuD *genes was shown to confer long-term protection against challenge with virulent *M. tuberculosis *in guinea pig model which is equivalent to that afforded by BCG [7]. Simian immunodeficiency virus (SIV)-positive and SIV-negative Rhesus macaques were immunized with *panCD *and *leuD *mutant strain, and safety studies, clinical, hematological and bacteriological monitoring were carried out and revealed no vaccine-associated adverse effects [7].

Another successful approach employed to rationally attenuate *M. tuberculosis *combines deletions in *secA2 *and *lysA *genes. The *secA2 *gene encodes a component of a virulence-associated bacterial protein secretion system involved in inhibiting the host immune system and consequently promoting the *M. tuberculosis *survival within the host. Thus, *secA2 *mutant was shown to increase both host cell apoptosis and priming of antigen-specific CD8^+ ^T cells *in vivo*. The *secA2 *and *lysA *double-mutant strain retained the effects obtained by *secA2 *single mutation, but with an improved safety profile in immunosuppressed mice [8].

Recently, the first live-attenuated *M. tuberculosis*-based vaccine, MTBVAC, entered clinical trials. MTBVAC contains in two independent deletions without antibiotic-resistance markers in the genes *phoP*, coding for a transcription factor key for the regulation of *M. tuberculosis *virulence, and *fadD26*, coding for one of the major mycobacterial virulence factors [9]. First, a mutant strain containing a single mutation in *phoP *gene was constructed and showed a high degree of safety, improved immunogenicity and protective efficacy compared to BCG in several animal models, from mice to non-human primates. Then, a second independent mutation deleting *fadD26 *gene was introduced to obtain MTBVAC which was shown to be functionally and phenotypically comparable to its prototype after rigorous preclinical safety and biodistribution experiments [9]. This vaccine candidate was genetically engineered to fulfill the Geneva consensus requirements for Phase I clinical trials of live mycobacterial vaccines candidates which demands improved protective efficacy and safety potential relative to BCG, and two non-reverting independent mutations without antibiotic resistance markers [10].

Overall, these multiple deletion mutants demonstrated the feasibility to obtain rationally attenuated *M. tuberculosis *strains, which so far were shown to be safe without compromising their ability to provide protective immunity, making them viable live vaccines candidates against TB.
